# Rapid and Durable Complete Remission of Refractory AITL with Azacitidine Treatment in Absence of TET2 Mutation or Concurrent MDS

**DOI:** 10.1097/HS9.0000000000000187

**Published:** 2019-03-01

**Authors:** Gareth P. Gregory, Michael Dickinson, Costas K. Yannakou, Jonathan Wong, Piers Blombery, Greg Corboy, Lev Kats, Tim M.E. Crozier, Beena Kumar, H. Miles Prince, Stephen S. Opat, Jake Shortt

**Affiliations:** 1 Monash Health, Clayton, Victoria, Australia; 2 School of Clinical Sciences at Monash Health, Monash University, Clayton, Victoria, Australia; 3 Clinical Haematology, Peter MacCallum Cancer Centre and Royal Melbourne Hospital, Parkville, Victoria, Australia; 4 Sir Peter MacCallum Department of Oncology, University of Melbourne, Parkville, Victoria, Australia; 5 Department of Molecular Oncology and Cancer Immunology, Epworth Healthcare, East Melbourne, Victoria, Australia; 6 Research Division, Peter MacCallum Cancer Centre, Parkville, Victoria, Australia.

Angioimmunoblastic T-cell lymphoma (AITL) is a rare disease entity associated with poor prognosis and no improvement in overall survival over the last 20 years.^1^ The genomic landscape of AITL has revealed frequent mutation of epigenetic modifiers *TET2* (76%), *DNMT3A* (33%) and *IDH2* (20%), genetic mutations that may be predictive of response to hypomethylating agents (HMA) in myelodysplastic syndromes.^2–4^ Genomic profiling has also demonstrated *TET2* mutations to be present in both malignant and non-malignant hematopoietic cells of affected individuals, suggesting loss of *TET2* to be the initiating mutation, following which secondary mutations direct the lineage phenotype of subsequent malignancy [eg, secondary *RHOA* mutations in AITL versus myeloid-lineage associated mutations in genes such as *RAS* leading to myelodysplasia (MDS) / chronic myelomonocytic leukemia (CMML)].^3,5^ The potential efficacy of HMAs in the treatment of AITL has emerged from the observation of regressing lymphadenopathy in patients treated for their concomitant MDS, however, such AITL responses may have been confounded by frequent concurrent rituximab administration for Epstein-Barr virus (EBV)-reactivation which is characteristic of this disease.^6–8^

Herein, we describe the case of a rapid, durable and complete response to azacitidine in a patient with AITL previously refractory to 10 lines of therapy. Next generation sequencing studies performed on the patient's tumor did not detect mutations or copy number alteration in recurrently mutated genes of AITL including *TET2*, *IDH2, RHOA* and/or *DNMT3A*. In addition, the patient did not have a concomitant diagnosis of MDS or receive treatment for EBV reactivation. We posit that the therapeutic benefit of azacitidine in AITL is not dependent on the presence of high-frequency recurrent mutations of canonical DNA methylation regulators, or at the very least that therapeutic benefit may still be derived in the absence of such mutations. These findings warrant prioritization of prospective studies of HMAs for patients with AITL irrespective of the mutational profile or presence of concomitant MDS.

**Figure 1:**
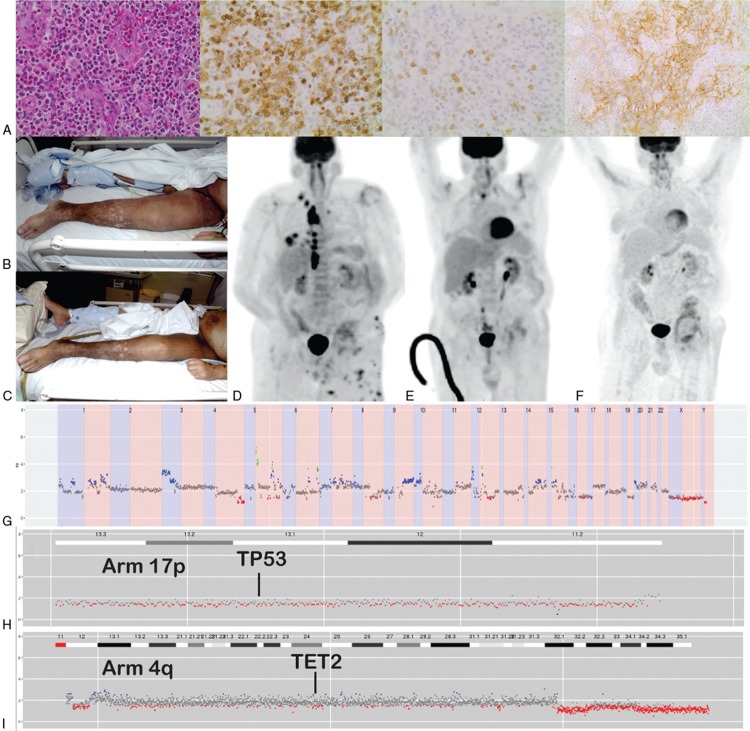
**Diagnostic, clinical and genomic aspects of the case**. (A) Diagnostic biopsy of the inguinal lymph node demonstrating an atypical lymphoid infiltrate and prominent high endothelial venules (far left, H&E stain 40x), positive staining of the lymphocytes for CD3 (inner left) and CD10 (inner right) and an irregular, expanded follicular dendritic cell population positively staining for CD21 (far right). (B) Clinical photography demonstrating lymphangitis and cutaneous disease prior to therapy on day 1 cycle 1, and (C) clinical response 11 days later. (D) Positron-emission tomography scan prior to azacitidine, (E) post-cycle three and (F) 3 years later demonstrating ongoing complete metabolic response. (G) Genome-wide copy number detection demonstrating significant copy number variation throughout the tumor genome. (H) Copy number detection demonstrating 17p monosomy. (I) Copy number detection demonstrating absence of significant copy number alteration affecting TET2.

A 54-year old man was referred to our service in 2011 with a rapidly enlarging left inguinal nodal mass and B-symptoms. Lymph node biopsy confirmed a diagnosis of AITL (Fig. 1A) and staging investigations confirmed Ann-Arbor stage II disease. The blood and bone marrow examinations exhibited marked eosinophilia, but no evidence of lymphoma, dysplasia or monocytosis. From 2011 to 2013, his disease was refractory to nine lines of therapy (Table 1) with only transient partial responses achieved at best. He was then enrolled in a phase II clinical trial of panobinostat (NCT01658241), however, he did not achieve an objective response to this agent. By 2014, the patient had received 10 lines of failed therapies in which he had never achieved a complete remission.

**Table 1:**
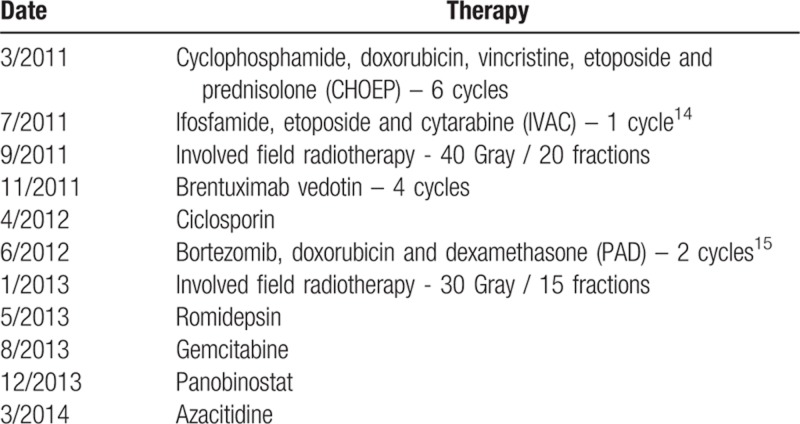
Therapies administered 2011 to 2018.

Given the emergent publications of the AITL genome, a rationale for re-purposing of hypomethylator therapy in this disease context was postulated.^2,3^ Following approval from our institutional Drug & Therapeutics Committee and with Australian TGA notification the patient consented to off-label treatment with azacitidine. Prior to commencement his disease had extranodal extension into the left femur with cutaneous involvement and extensive lymphedema due to tumor-related obstruction (Fig. 1B) and the patient was admitted to the intensive care unit following respiratory arrest secondary to viral pneumonitis (H1N1 and Influenza A) complicated by acute cardiomyopathy and polymicrobial chest sepsis for which he was requiring physiological support.

During the first cycle of azacitidine (75 mg/m^2^ by subcutaneous injection given days 1–7)^9^ the patient had a rapid response with regression of cutaneous lesions and resolution of lymphedema following large diuresis independent of altered cardiovascular status (Fig. 1C). He subsequently achieved a complete metabolic remission (CMR) after three cycles of therapy (Fig. 1D-E). He is currently in ongoing CMR after 60 cycles of azacitidine (Fig. 1F).

DNA was extracted from fresh cutaneous tissue obtained immediately prior to treatment with panobinostat and sequenced using a hybridization-based NGS panel covering approximately 300 genes recurrently mutated in hematological malignancy.^10^ A sequence variant in TP53 (NM_000546.5:c.376_394del, p.(Tyr126Argfs∗38)) predicted to result in a truncated protein product was detected. In addition a missense mutation was detected in NCOR1 (NM_001190440.1:c.4627G>A, p.(Glu1543Lys)). Analysis of whole genome copy number was performed using off-target reads as described previously.^10^ This revealed multiple copy number abnormalities throughout the genome (Fig. 1G) but specifically a copy number loss involving TP53 (Fig. 1H) and no significant copy number alteration affecting TET2 (Fig. 1I). Importantly, no candidate pathogenic mutations were detected in the entire coding regions of TET1, TET2, TET3, RHOA, DNMT3A, and IDH2. The percentage of the TET2 gene covered was 100% with a mean coverage of 486X and 99.43% of the gene covered >100X.

Next generation sequencing technologies have rapidly advanced our understanding of the genetic landscape of incurable hematological diseases such as AITL. The recognition of frequent somatic mutation of regulators of DNA methylation^2^ has revealed an unanticipated overlap with the molecular signature of MDS, an HMA-responsive disease. The co-occurrence of clonally-related MDS, in particular CMML, is increasingly recognized as an AITL disease association. Although the intra-clonal architecture has not been mapped at a single-cell level, it is postulated founder mutations of DNA methylation regulators may seed progeny with both MDS or T-cell lymphoma phenotypes, depending on the nature of secondary mutational events.^3^ This hypothesis is supported by a recent case report of an individual with AITL and acute myeloid leukemia, in which the relative variant allele frequencies in the pre-leukemic bone marrow and leukemic bone marrow suggested clonal evolution from a common founder mutation.^5^ From a clinical perspective, concurrent CMML may be unrecognized, due to the presumption of ‘reactive’ monocytosis in the context of immune dysregulation and the attribution of cytopenias to bone marrow infiltration by lymphoma. Indeed, the first reported case of an objective response to azacitidine in AITL was in a patient being treated primarily for CMML and with concurrent rituximab for EBV reactivation.^6^

The mechanism of action of HMAs in MDS remains incompletely understood. *TET2* mutations predict a marginally higher response rate to HMA, but significant clinical responses are also observed in the absence of *TET2* loss. The delay between rapid changes in DNA methylation and clinical remission (which may take 6 months) is potentially explained by secondary immune mediated effects.^11^ This hypothesis is supported by evidence from recent studies of *DUSP22*-rearranged anaplastic large cell lymphoma wherein the hypomethylated molecular signature was associated with an immunogenic phenotype.^12^ Furthermore, peripheral T-cell lymphomas including AITL have recently been demonstrated to have a consistent methylation immunophenotype with absence of 5-hydroxymethylcytosine in malignant cells irrespective of the mutational profile including *TET2* mutational status, and it remains unclear as to whether this may confer specific sensitivity to HMAs.^13^

The present case is remarkable as the patient had disease that was refractory to multiple conventional and novel therapies and yet the response to azacitidine occurred within days of exposure and has been maintained for years thereafter in the absence of the somatic mutational profile we assumed was being ‘targeted’ (ie, *TET2 or IDH2* mutant disease). This suggests an unanticipated synthetic-lethal interaction with lymphoma biology that is unrelated to the hypothesis-based application of an HMA and also independent of direct cytotoxicity in the setting of bi-allelic TP53 disruption. The tempo of response is also of interest as the total cellularity of an involved node/tissue with AITL is largely contributed by reactive cells in the microenvironment, and it is possible that the rapid clinical regression is an effect of HMA therapy on the microenvironment rather than purely cell-intrinsic to the malignant cells.

This case is noteworthy for genomic instability as evidenced by the complex CNV changes, and further studies assessing efficacy of HMA therapy in this setting may be warranted. In light of recent retrospective data suggesting a 75% response rate to azacitidine in AITL,^8^ further prospective evaluation of HMAs in T-cell lymphoma is imminent. Our exceptional responder indicates that mutational profiling, in particular *TET2* status, should not be the basis of pre-selection for such studies and that even highly refractory disease can demonstrate dramatic and durable responses.
